# Fulfillment and Validity of the Kidney Health Evaluation Measure for People with Diabetes

**DOI:** 10.1016/j.mayocpiqo.2023.07.002

**Published:** 2023-08-29

**Authors:** Silvia Ferrè, Amy Storfer-Isser, Kelsy Kinderknecht, Elizabeth Montgomery, Miriam Godwin, Ashby Andrews, Stephan Dunning, Mary Barton, Dan Roman, John Cuddeback, Nikita Stempniewicz, Chi D. Chu, Delphine S. Tuot, Joseph A. Vassalotti

**Affiliations:** a National Kidney Foundation, New York, NY; b National Committee for Quality Assurance, Washington, DC; c Optum Labs, Eden Prairie, MN; d American Medical Group Association, Alexandria, VA; e University of California at San Francisco, San Francisco, CA; f Department of Medicine, Division of Nephrology, Icahn School of Medicine at Mount Sinai, New York, NY

## Abstract

**Objective:**

To evaluate the fulfillment and validity of the kidney health evaluation for people with diabetes (KED) Healthcare Effectiveness Data Information Set (HEDIS) measure.

**Patients and Methods:**

Optum Labs Data Warehouse (OLDW) was used to identify the nationally distributed US population aged 18 years and older, with diabetes, between January 1, 2017, and December 31, 2017. The OLDW includes deidentified medical, pharmacy, laboratory, and electronic health record (EHR) data. The KED fulfillment was defined in 2017 as both estimated glomerular filtration rate (eGFR) and urinary albumin-creatinine ratio testing within the measurement year. The KED validity was assessed using bivariate analyses of KED fulfillment with diabetes care measures in 2017 and chronic kidney disease (CKD) diagnosis and evidence-based kidney protective interventions in 2018.

**Results:**

Among eligible 5,635,619 Medicare fee-for-service beneficiaries, 736,875 Medicare advantage (MA) beneficiaries, and 660,987 commercial patients, KED fulfillment was 32.2%, 38.7%, and 37.7%, respectively. Albuminuria testing limited KED fulfillment with urinary albumin-creatinine ratio testing (<40%) and eGFR testing (>90%). The KED fulfillment was positively associated with receipt of diabetes care in 2017, CKD diagnosis in 2018, and evidence-based kidney protective interventions in 2018. The KED fulfillment trended lower for Black race, Medicare-Medicaid dual eligibility status, low neighborhood income, and low education status.

**Conclusion:**

Less than 40% of adults with diabetes received guideline-recommended testing for CKD in 2017. Routine KED was associated with diabetes care and evidence-based CKD interventions. Increasing guideline-recommended testing for CKD among people with diabetes should lead to timely and equitable CKD detection and treatment.

Diabetes affects more than 34 million US adults and is the leading cause of chronic kidney disease (CKD),^1^ occurring in 30%-40% of patients with diabetes.^2^ Chronic kidney disease is characterized by poor outcomes, such as cardiovascular disease, mortality, and progression to end-stage kidney disease, requiring therapy with dialysis or kidney transplant.^3^, ^4^, ^5^ Together, CKD and end-stage kidney disease impose a high financial burden, accounting for over $133 billion in 2019 Medicare costs.^6^

Despite the high prevalence and complication burden of CKD, ∼90% of people with CKD in the United States are unaware of their condition. This fact can be attributed to the asymptomatic nature of the condition, under-testing, and low diagnosis rates, even among high-risk groups.^7^, ^8^, ^9^, ^10^, ^11^, ^12^, ^13^ Clinical practice guidelines from the American Diabetes Association (ADA) and the National Kidney Foundation recommend testing patients with diabetes for kidney disease at least annually using both the estimated glomerular filtration rate (eGFR) on the basis of serum creatinine to assess kidney function and urine albumin-creatinine ratio (uACR) to detect kidney damage or endothelial inflammation.^2^, ^3^, ^4^, ^5^ Health services research shows that fewer than half of adults with diabetes receive annual eGFR and uACR testing, with low albuminuria testing as the primary limitation.^10^, ^11^, ^12^, ^13^

Risk stratification on the basis of both eGFR and uACR guides evidence-based interventions.^2^, ^3^, ^4^, ^5^, ^7^, ^8^, ^14^, ^15^, ^16^, ^17^ The ADA recommends treatment with angiotensin converting enzyme inhibitors (ACEi) or angiotensin II receptor blockers (ARB) in all patients with diabetes and hypertension when uACR ≥30 mg/g or eGFR is <60 mL/min/1.73 m^2^. For people with type 2 diabetes, ADA recommends sodium–glucose cotransporter 2 inhibitor (SGLT2i) initiation when eGFR is ≥20 mL/min/1.73 m^2^ and uACR is >200 mg/g.^18^ Finally, the severity of eGFR reduction or uACR increase informs recommendations for interdisciplinary care with a registered dietitian or nephrologist.^2^, ^3^, ^4^, ^5^, ^14^, ^16^, ^17^, ^18^, ^19^, ^20^

The previously applicable HEDIS medical attention for nephropathy (MAN) measure is problematic. First, numerator satisfaction can occur in any of 4 ways, such as any urine protein or albumin test, use of diagnosis or procedure codes for CKD, visit with a nephrologist, prescription of ACEi or ARB. Second, the measure was satisfied by many health insurance plans, masking any existing gaps in care.^21^, ^22^ Third, an evaluation of the MAN measure found ACEi or ARB therapy contributed to 14%-16% of the numerator satisfaction, bringing the final fulfillment rate to above the 80% range.^22^ However, only 1% of the patients satisfying the numerator this way had the uACR test in previous 5 years and none in the reporting year.^22^ The authors concluded that the use of ACEi or ARB does not obviate the need for uACR screening, and the inclusion of the medications in the measure numerator leads to overreporting and may contribute to underscreening of the population with diabetes at risk.^22^ To address this gap in care and advance guideline-concordant testing in the United States, National Kidney Foundation and the National Committee for Quality Assurance developed the KED measure and collaborated on this retrospective population assessment of the KED measure.

## Methods

### Study Design and Data Sources

This retrospective cohort study was conducted using OLDW data^23^ that contains deidentified, longitudinal medical and pharmacy claims and enrollment data with linked laboratory results, socioeconomic status, and death information for a sample of commercial, MA, and Medicare FFS enrollees. Neighborhood education and income data were derived from the 2014 to 2019 American community survey 5-year sample and attributed to cohort members at the zip code tabulation area level. Race and ethnicity data for MA and Medicare FFS enrollees were ascertained from the Medicare master beneficiary summary file.^24^ Medicare FFS claims files held by Optum Labs, a certified Centers for Medicare and Medicaid Services qualified entity, were approved for re-use by Centers for Medicare and Medicaid Services. Because this study involved the analysis of preexisting deidentified data, the New England institutional review board considered this study exempt from review.

### Study Population

Medicare FFS, MA, and commercial patients, aged 18 years and older as of December 31, 2017, who were continuously enrolled (no more than 1 gap of up to 45 days) in medical benefits between January 1, 2017 and December 31, 2017, were identified. The KED measure fulfillment was determined see Table 1. Briefly, the eligible population (denominator) included people who were identified with type 1 or type 2 diabetes using medical and pharmacy claims data per the HEDIS. Although the measure specification defines the eligible population as aged 18-85 years, this analysis included all beneficiaries aged 18 years or over to explore KED fulfillment for all adults. The measure numerator included patients who received at least 1 eGFR test and at least 1 uACR test, during the measurement year.

**Table 1:** Kidney Health Evaluation for patients with Diabetes Measure Description

Numerator	Patients who received a kidney health evaluation, defined by an eGFR and a uACR, during the measurement year are as follows: • At least 1 eGFR is required during the measurement period • At least 1 uACR is required during the measurement period The uACR is identified by the member having both a quantitative urine albumin test and aurine creatinine test
Denominator (Eligible population)	All beneficiaries aged 18-85 years with type 1 or type 2 diabetes who had any of the following: • At least 2 outpatient visits, observation visits, telephone visits, e-visits, or virtual check-ins, or nonacute inpatient encounters or discharges with a diagnosis of diabetes in the measurement year or year before the measurement year • At least 1 acute inpatient encounter or discharge with a diagnosis of diabetes in the measurement year or year before the measurement year • At least 1 ambulatory pharmacy claims for medications specific to diabetes treatment in the measurement year or year before the measurement year
Exclusions	End-stage kidney disease, dialysis, diagnosed CKD stage 5 by ICD-10-CM N18.5, palliative care, hospice, enrollment in Institutional Special Needs Plan (Medicare advantage only), frailty or advanced illness^25^

Abbreviations: CKD, chronic kidney disease; eGFR, estimated glomerular filtration rate; uACR, urine albumin-creatinine ratio.

### Outcomes

The primary outcome was KED fulfillment. To assess KED measure validity, we calculated fulfillment with 3 HEDIS comprehensive diabetes care measures in 2017: MAN,^21^, ^22^ receipt of an HbA1c test, and receipt of a retinal eye exam.^25^ To further assess validity, we identified people with a CKD diagnosis in 2018 and received evidence-based interventions for CKD in 2018. Nonpharmacological kidney protective interventions in 2018 included medical nutrition therapy and a nephrology consultation. Pharmacological CKD interventions in 2018 included kidney and cardiovascular protective classes of hypertension medications (ACEi or ARB), statin use in persons with diabetes (HEDIS measure),^25^^,^^26^ and kidney and cardiovascular protective type-2 diabetes medication class (SGLT2 inhibitor) use. Finally, we examined HbA1c control (<8%)^8^ and blood pressure (BP) control (<140 systolic and <90 diastolic mmHg) in 2018.

### Statistical Analyses

All analyses were stratified by insurance type. Descriptive statistics included counts and percentages for categorical variables, means and standard deviations for normally distributed variables, and medians, 25th and 75th percentiles for skewed variables. Validity of the KED measure was assessed using bivariate analyses. Agreement between the KED and the MAN measures was assessed using the κ coefficient. Spearman’s correlation was used to examine the association of the KED measure with each of the comprehensive diabetes care measures. To further assess the validity, 4 KED groups were created, as follows: (1) KED fulfilled (received both eGFR and uACR tests); (2) received eGFR but not uACR; (3) received uACR but not eGFR; and (4) those who received neither test. For each group, we summarized the percentage of patients who fulfilled each of the 3 comprehensive diabetes care measures, reported a CKD diagnosis code in 2018, and received evidence-based interventions in 2018. Data cleaning, statistical analysis, and visualization were performed using SAS.

## Results

After applying the inclusion and exclusion criteria (Table 1 and Supplemental Table 1, available online at http://www.mcpiqojournal.org), the study population included 5,635,619 Medicare FFS, 736,875 MA, and 660,987 commercial patients eligible for the KED measure with characteristics shown in Table 2. Medicare patients were older than commercial patients. About half of Medicare FFS and MA patients were woman, and 44% of commercial patients were woman. Although most of the eligible population was non-Hispanic or Latino White across all 3 types of insurance, MA beneficiaries found more non-Hispanic or Latino Black or African American beneficiaries (20%) compared with the Medicare FFS (13%). About 1 in 4 Medicare FFS beneficiaries (24%) and 1 in 5 Medicare advantage beneficiaries (21%) were dually eligible for Medicare and Medicaid services. Across all 3 insurance types, about half of beneficiaries lived in the Southern United States.

**Table 2:** Characteristics of the Population Eligible for the KED Measure in 2017 Stratified by Insurance Type

Numerator	Denomiator					
HEDIS Measure	Medicare FFS (n=5,635,619)		MA (n=736,875)		Commercial (n=660,987)	
	n	(%)	n	(%)	n	(%)
KED	1,813,100	32.2	285,017	38.7	249,316	37.7
MAN	4,925,071	87.4	681,946	92.5	549,351	83.1
HbA1c test	4,957,572	88.0	683,591	92.8	592,183	89.6
Retinal eye exam	3,507,473	62.2	468,320	63.6	242,255	36.7

Abbreviations: FFS, fee-for service; HbA1c: hemoglobin A1c; KED, kidney health evaluation for people with diabetes; MAN: medical attention for nephropathy; MA, Medicare advantage

### KED Fulfillment and Comparison with Other Quality Measures

The KED fulfillment was below 40% among the 3 insurance types: Medicare FFS (32.2%), MA (38.7%), and commercial (37.7%) beneficiaries (Figure). Although more than 90% of the beneficiaries received an eGFR test, less than 40% had a uACR test (Figure). Less than 10% of the beneficiaries had neither an eGFR nor a uACR test. Less than 1% of the beneficiaries had a uACR test but did not have an eGFR test; given this result, this group was not included in subsequent analyses.

**Figure:**
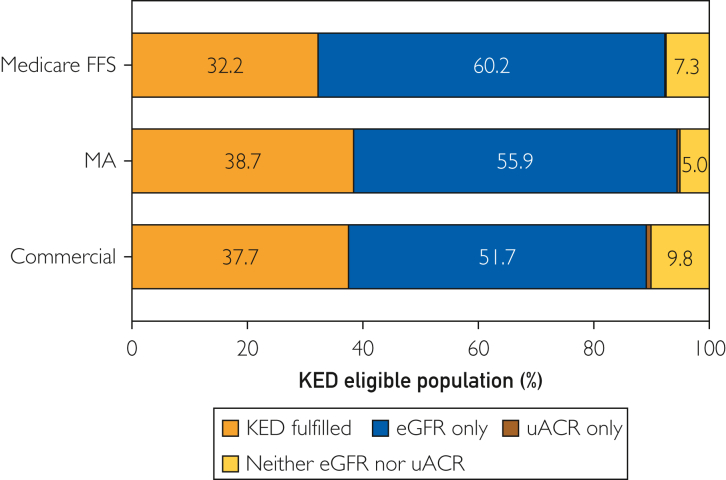
Use of CKD tests by health insurer. KED numerator fulfillment breakdown by test type across insurance types. KED, kidney health evaluation for people with diabetes; FFS, fee-for service; eGFR, estimated glomerular filtration rate; uACR, urine albumin-creatinine ratio.

Fulfillment was high for the MAN measure in 87.4% of Medicare FFS, 92.5% of MA, and 83.1% of commercial populations (Supplemental Table 2, available online at http://www.mcpiqojournal.org). Agreement between the KED and MAN measures was *κ*=0.128 for Medicare FFS, *κ*=0.097 for MA, and *κ*=0.219 for commercial beneficiaries.

Spearman correlations between the KED and HbA1c testing measures were *r*=0.26 for Medicare FFS, *r*=0.23 for MA, and *r*=0.35 for commercial beneficiaries. Across the 3 insurance types, fulfillment of the KED measure was associated with higher HbA1c testing and retinal eye exam measures (Supplemental Figure, available online at http://www.mcpiqojournal.org). Approximately 99% of beneficiaries with KED measures fulfilled had HbA1c testing, whereas 90%-93% of beneficiaries with only an eGFR and only 28%-36% of beneficiaries receiving neither test had HbA1c testing (Supplemental Figure). Spearman correlations between the KED and retinal eye exam measures were *r*=0.12 for Medicare FFS and *r*=0.11 for MA and commercial beneficiaries. The percentage of beneficiaries with the retinal eye exam measure fulfilled was highest among those who had the KED measure fulfilled (43%-70%) compared with beneficiaries with only an eGFR (34%-61%) and beneficiaries receiving neither test (25%-44%)

### KED Fulfillment and Demographic Characteristic Factors

The KED measure fulfillment in 2017 in the Medicare population was similar between ages 65-75 years and 76-85 years, with slightly lower KED performance in the older age group (Table 3). The difference between performances in those same 2 age bands within the commercial population is noticeably larger; however, substantially fewer people in the 76 to 85 years age group (less than 1% of the commercial population). With respect to race and ethnicity (Table 3), fulfillment of the KED measure in the Medicare population was highest among Asian American beneficiaries (43%-48%) and lowest among Black or African American beneficiaries (30%-36%). Fulfillment of the KED measure was lower among those dually eligible for Medicare and Medicaid compared with the nondual eligible population (33%-40% vs 29%-35%, respectively; Table 3). The completion of the KED measure increased as neighborhood income increased across all 3 health insurance populations (Table 3). Similarly, fulfillment of the KED measure increased as neighborhood education increased, on the basis of the percent of the neighborhood with a bachelor’s degree or higher (Table 3).

**Table 3:** KED Fulfillment In 2017 Stratified by Demographic Characteristic Factors

Demographic Characteristic Factor	Medicare FFS	MA	Commercial
Age range (y)			
18-64	26.9	34.4	37.8
65-75	34.3	40.5	38.4
76-85	32.6	38.5	25.3
Race and ethnicity			
White	31.6	37.4	N/A
Black/African American	29.5	35.7	N/A
Hispanic/Latino	35.2	48.4	N/A
Asian American	42.6	48.2	N/A
Dual eligibility			
Yes	28.9	35.2	N/A
No	33.2	39.6	N/A
Neighborhood income range ($)			
<$40,750	25.7	34.8	31.7
$40,750 to <$73,245	31.8	38.8	36.1
≥$73,245	37.9	42.8	40.6
Neighborhood with bachelor’s degree or higher			
Low	27.1	35.4	33.0
Moderate	31.0	37.8	35.7
High	37.0	42.8	40.2

Abbreviations: FFS, fee-for service; KED, kidney health evaluation for people with diabetes; MA, Medicare advantage; N/A, not applicable.

### KED Fulfillment and Guideline-Indicated Care

Beneficiaries with the KED measure in 2017 fulfilled were more likely to have a CKD diagnosis in 2018 (7.4%-26.7% in KED fulfilled beneficiaries compared with 3.5%-11.8% in beneficiaries who received neither test) (Table 4 and Supplemental Table 3, available online at http://www.mcpiqojournal.org). Across all insurance plans, beneficiaries with the KED measure in 2017 fulfilled were more likely to receive nonpharmacological evidence-based interventions for CKD in 2018 than those who did not have the KED measure fulfilled. Nonpharmacological interventions for CKD included medical nutrition therapy for CKD or diabetes (6.0%-7.7% in KED fulfilled beneficiaries compared with 3.4%-4.0% in beneficiaries who received neither test) and nephrology consultation (4%-11% compared with 1.7%-3.7%) (Table 4). Across all insurance plans, beneficiaries with the KED measure fulfilled in 2017 were also more likely to receive pharmacological evidence-based interventions for CKD in 2018 than those who did not have the KED measure fulfilled. Pharmacological evidence-based interventions for CKD included ACEi or ARB therapy (64.8%-75.4% in KED fulfilled beneficiaries compared with 44.4%-59.3% in beneficiaries who received neither test), statin therapy (66.8%-75.6% compared with 45.0%-54.3%), and SGLT2i therapy (5.7%-17.7% compared with 2.9%-9.7%) (Table 4). Completion of the KED measure in 2017 was associated with better HbA1c control (<8%) and BP control (<140/90 mmHg) in 2018. Of importance, the percentage of beneficiaries who received interventions for CKD in 2018 was similar or higher among beneficiaries with KED measure fulfilled compared with those who had MAN measure fulfilled in 2017 (Table 4).

**Table 4:** KED and MAN Fulfillment in 2017 and Guideline-Indicated Care in 2018 Stratified by Insurance Type

Outcome in 2018	Medicare FFS				MA				Commercial			
	MAN Fulfilled	KED Fulfilled	eGFR Only	Neither eGFR Nor uACR	MAN Fulfilled	KED Fulfilled	eGFR Only	Neither eGFR Nor uACR	MAN Fulfilled	KED Fulfilled	eGFR Only	Neither eGFR Nor uACR
CKD diagnosis (%)	23.8	25.5	20.8	11.1	24.2	26.7	21.3	11.8	7.4	7.4	6.3	3.5
Medical nutrition therapy (%)	6.7	7.7	5.9	4.0	5.5	6.0	5.0	3.4	5.7	6.1	5.3	3.9
Nephrology consultation (%)	9.9	11.0	8.4	3.7	9.9	10.7	8.8	3.7	3.9	4.0	3.4	1.7
ACEi/ARB therapy (%)	74.8	74.6	68.6	59.3	75.7	75.4	70.7	56.2	66.8	64.8	60.2	44.4
SGLT2 inhibitor therapy (%)	5.4	6.4	4.8	3.7	5.1	5.7	4.6	2.9	15.9	17.7	14.2	9.7
Statin therapy (Age 40+, %)	91.2	75.4	67.7	54.3	72.4	75.6	68.9	51.3	63.1	66.8	57.9	45.0
HbA1c control (HbA1c <8%) rate	64.1	73.7	56.3	29.0	76.8	79.1	75.1	60.1	66.8	68.8	66.0	48.1
BP control (%)	65.4	69.0	62.8	38.1	70.7	73.2	68.6	51.3	71.8	74.8	68.3	56.0

Abbreviations: ACEi, angiotensin converting enzyme inhibitor; ARB, angiotensin II receptor blocker; CKD, chronic kidney disease; eGFR, estimated glomerular filtration rate; FFS, fee-for service; KED, kidney health evaluation for people with diabetes;; MA, Medicare advantage; MAN: medical attention for nephropathy; SGLT2 inhibitor, sodium–glucose cotransporter-2 inhibitor; uACR, urine albumin-creatinine ratio.

### Exploratory Analyses

We conducted a series of exploratory analyses to better understand nonfulfillment with uACR testing. Approximately 17%-20% of the beneficiaries who did not have uACR fulfillment had a quantitative urine albumin test but did not have a urine creatinine test, whereas 7%-8% had a urine creatinine test but did not have a quantitative urine albumin test.

## Discussion

This retrospective study, including a large and diverse sample of Medicare FFS, MA, and commercially insured people with diabetes, aged 18 years and older, supports the validity of the new HEDIS KED measure. The results found that KED fulfillment in 2017 was positively correlated with 3 HEDIS comprehensive diabetes care measures. Furthermore, KED fulfillment in 2017 was associated with CKD diagnosis, interdisciplinary care, and evidence-based kidney protective pharmacologic interventions during the following year. In this analysis, between 78%-90% of beneficiaries, whose KED measure was incomplete owing to the eGFR test only had MAN measure fulfilled. This confirms that the MAN measure may lead to overreporting and may contribute to underscreening, particularly with uACR and undertreatment of high-risk populations (Supplemental Graphical Abstract, available online at http://www.mcpiqojournal.org).^22^

However, fulfillment with the KED measure was under 40% for the population with diabetes who should be receiving annual eGFR and uACR testing according to clinical practice guideline recommendations.^8^^,^^18^ In particular, absent uACR testing contributed to lower performance on the KED measure, although greater than 90 percent of the diabetes population received an annual eGFR test. These findings were consistent across Medicare FFS, MA, and commercial insurance populations. Approximately 40% or less annual uACR testing for the US diabetes population was shown in other national datasets from the 5% Medicare sample,^27^ clinical laboratories,^11^ and health systems,^12^ supporting the need for interventions and quality improvement measures to increase targeted albuminuria testing.^10^, ^11^, ^12^^,^^28^ In addition, further analysis of the subpopulation without a uACR test found that 17%-20% had a quantitative urine albumin test, whereas 7%-8% had a urine creatinine test. This may suggest a knowledge gap of clinicians regarding the recommended uACR test order or that some laboratories may not offer the uACR result at all, offering only the urine albumin concentration, or alternatively require clinicians to order urine albumin and urine creatinine separately. Thus, clinicians may have the intent to test for uACR but do not receive credit for the testing because the laboratory does not report the ratio, emphasizing the need for laboratories to facilitate uACR ordering and reporting in mg/g, as recommended by clinical practice guidelines.^29^^,^^30^

Analysis of several intermediate outcomes in 2018 reported that beneficiaries with the KED measures fulfilled in 2017 were more likely to be diagnosed with CKD, receive medical nutrition therapy, and receive nephrology consultation in 2018 than those with only an eGFR test or those with neither test in 2017. Furthermore, beneficiaries with the KED measure fulfilled were more likely to receive evidence-based kidney protective medications in 2018 than persons whose measure was incomplete, such as ACEi or ARB therapy^31^, ^32^, ^33^ and SGLT2i therapy.^34^^,^^35^ The SGLT2i therapy was low across all insurance types; prescription of these drugs by clinicians in the United States remains low despite their kidney protective effects and studies supporting cost-effectiveness.^36^^,^^37^ Of importance, SGLT2 inhibitor therapy for CKD was likely underestimated in this study because the entire patient population with type 2 diabetes was included rather than patients with type 2 diabetes, eGFR ≥20 mL/min/1.73 m^2^ and uACR >200 mg/g, which are the kidney disease indications for SGLT2i use according to current ADA recommendations.^18^ Moreover, study period evidence for SGLT2i use in CKD should be interpreted with the caveat that the 3 seminal randomized placebo-controlled trials with primary kidney outcomes were all published subsequently, between 2019 and 2023. In this study, we also found that patients with the KED measure fulfillment vs those without completion in 2017 were more likely to have BP and diabetes control.

We found that there are disparities in kidney health evaluation testing, with lower KED fulfillment among Black or African Americans and people with Medicare-Medicaid dual eligibility, lower neighborhood income, and lower education status. The KED measure fulfillment was highest among Asian American beneficiaries (43%-48%) followed by Hispanic or Latino (35%-48%), White (32%-37%), and Black or African American (30%-36%). These findings are novel with the results from other studies showing higher albuminuria testing or CKD care process measures in Asian Americans with diabetes or CKD, respectively, compared with White, Black, and Hispanic patients.^13^^,^^19^ This may be explained by greater trust in the health care system among Asian Americans, greater clinician testing, and increased patient-clinician encounters.^13^ The Black or African American and Hispanic or Latino populations have a disproportionately higher burden of diabetes, advanced kidney disease, and cardiovascular disease; however, CKD testing in these groups is low. As a result of structural racism, Black or African American and Hispanic or Latino individuals are disproportionately affected by poverty and experience more food insecurity than White individuals.^28^ Several studies have correlated lower socioeconomic status with a higher prevalence of diabetes and CKD.^28^^,^^38^ Along with lower income, Americans with fewer than 12 years of education were also found to show a greater prevalence of diabetes and lower kidney function.^28^^,^^38^ In addition, inequities in care delivery on the basis of race, sex, and socioeconomic status have been observed.^39^, ^40^, ^41^, ^42^, ^43^, ^44^ Higher KED measure performance across all populations leads to higher rates of multidisciplinary care, and evidence-based interventions may help reduce racial or ethnic and socioeconomic disparities in clinical outcomes among individuals with diabetes and CKD. However, similar disparities are also observed in the use of novel pharmacologic agents, such as SGLT2i agents, that are prescribed less in Black or African American patients and patients with lower socioeconomic status.^44^ Barriers to the adoption of novel therapeutic agents include decreased access to quality diabetes care and to specialists familiar with the new agents; structural racism; clinician bias that certain groups of patients may be less likely to adhere to treatment with an expensive agent; and prescription abandonment owing to economic barriers.^40^^,^^42^^,^^43^ Interventions to ensure more equitable kidney health evaluation and the use of effective treatments are essential to preventing the worsening of well-documented disparities in kidney and cardiovascular outcomes among patients with diabetes in the United States.

Strengths of this study are the large sample size and variety of data sources that permitted the description of CKD testing in populations across commercial and Medicare health insurance, diverse neighborhood incomes, education status, and identification with racial and ethnic groups. However, limitations should also be acknowledged. First, eGFR and uACR testing that occur without claims data might not be captured, potentially leading to underreporting of testing rates. Second, serum creatinine for eGFR is included in testing panels that are a routine part of clinical care for adults with acute and chronic medical conditions other than type 2 diabetes and thus may not reflect a deliberate kidney health evaluation. Third, race and ethnicity may not always represent self-reported race and may not capture multiracial or multiethnic status. Furthermore, race and ethnicity data were not included for commercial patients because of incomplete data. Fourth, we did not analyze the association between KED fulfillment and cardiovascular outcomes or death, owing to the assessment period of only one year.^45^ Fifth, although the study suggests that increasing guideline-recommended testing for CKD among people with diabetes may be an important first step toward timely and equitable CKD treatment, a pragmatic randomized clinical trial coupled with professional education and real-world evidence generation strategies is needed to show whether CKD testing leads to increased CKD treatment.

In conclusion, this retrospective study using a US national database of medical and pharmacy claims, EHR data, and claims-based laboratory test results supports the validity of the KED measure for adults with diabetes and highlights the importance of routine kidney health evaluation in high-risk populations. A screened population should have greater awareness of CKD, be better educated and managed, and be better prepared for kidney failure should that occur.

The KED measure is currently part of HEDIS, replacing the existing HEDIS MAN quality measure.^21^ The long-term goal is to support the effort to connect the KED measure for implementation in both federal, Merit-based Incentive Payment System and commercial health insurance quality measure programs.

## Potential Competing Interests

J.A.V. reports honoraria from Renalytix, PLC. (Advisory Board) and Astra Zenica, Inc. (consultant).
