# Supplementary material for: Fulfillment and Validity of the Kidney Health Evaluation Measure for People with Diabetes
Source: Mayo Clin Proc Innov Qual Outcomes. 2023 Aug 29;7(5):382–91. doi: 10.1016/j.mayocpiqo.2023.07.002 (PMC10480072; doi:10.1016/j.mayocpiqo.2023.07.002)

Supplemental Materials

Table S1. Identification of the population eligible for the KED measure in 2017 stratified by insurance type.

	Medicare FFS		MA		Commercial	
**Initial Population (N, %)***	**7,674,102**	**(100)**	**930,144**	**(100)**	**757,678**	**(100)**
Excluded due to Medicare as primary insurance (commercial beneficiaries only)	n/a		n/a		87,164	(11.5)
Required medical exclusions	2,038,483	(27)	193,269	(21)	16,527	(19)
o Hospice	27,363	(0.4)	694	(0.1)	247	(0.03)
o Palliative Care	69,350	(0.9)	5,563	(0.6)	553	(0.07)
o End Stage Renal Disease (ESRD) or Dialysis	343,789	(4.5)	23,170	(2.5)	4,426	(0.6)
o Institutional Special Needs Plan (I-SNP)**	n/a		11,069	(1.2)	n/a	
o Frailty/Advanced illness	1,597,981	(20.8)	152,773	(16.4)	4,301	(0.6)
**KED Eligible Population (N, %)**	**5,635,619**	**(100)**	**736,875**	**(100)**	**660,987**	**(100)**

* Continuously enrolled in medical benefits in 2017, age 18+ years as of December 2017, meet KED HEDIS^{TM} criteria for diabetes.

** Exclusion applies to Medicare Advantage population age 66+ years only.

Abbreviations: KED, kidney health evaluation for people with diabetes; FFS, fee-for service; MA, Medicare Advantage.

Table S2. Fulfillment on the KED, MAN, HbA1c test, and retinal eye exam measures in 2017.

		Denominator					
	HEDIS™ measure	Medicare FFS (N=5,635,619)		MA (N=736,875)		Commercial (N=660,987)	
		N	%	N	%	N	%
Numerator	KED	1,813,100	32.2	285,017	38.7	249,316	37.7
	MAN	4,925,071	87.4	681,946	92.5	549,351	83.1
	HbA1c test	4,957,572	88.0	683,591	92.8	592,183	89.6
	Retinal eye exam	3,507,473	62.2	468,320	63.6	242,255	36.7

Abbreviations: KED, kidney health evaluation for people with diabetes; MAN: medical attention for nephropathy; FFS, fee-for service; MA, Medicare Advantage; HbA1c: hemoglobin A1c

Table S3. Eligible beneficiaries with continuous enrollment in 2017 and 2018 and assessed evidence-based interventions in 2018.

Outcome in 2018	Limitation in 2018	Medicare FFS (N=5,119,274; 100%)		MA (N=643,507; 100%)		Commercial (N=493,416; 100%)	
		N	%	N	%	N	%
CKD diagnosis	Medical benefit	1,126,240	22	148,006	23	29,604	6
Medical nutrition therapy	Medical benefit	307,156	6	32,175	5	29,604	6
Nephrology consultation	Medical benefit	460,734	9	57,915	9	1,479	3
**Outcome in 2018**	**Limitation in 2018**	**(N=3,772,458; 100%)**		**(N=561,333; 100%)**		**(N=277,557; 100%)**	
		**N**	**%**	**N**	**%**	**N**	**%**
ACEi/ARB therapy	Medical and pharmacy benefits	2,640,720	70	404,159	72	166,534	60
SGLT2 inhibitor therapy	Medical and pharmacy benefits	188,622	5	28,066	5	41,633	15
**Outcome in 2018**	**Limitation in 2018**	**(N=2,624,370; 100%)**		**(N=417,994; 100%)**		**(N=217,046; 100%)**	
		**N**	**%**	**N**	**%**	**N**	**%**
Statin therapy (Age 40+)*	Medical and pharmacy benefits, age, and medical exclusions	1,823,235	69	296,775	71	130,227	60
**Outcome in 2018**	**Limitation in 2018**	**(N=266,685; 100%)**		**(N=353,798; 100%)**		**(N=249,622; 100%)**	
		**N**	**%**	**N**	**%**	**N**	**%**
HbA1c control	Medical benefit, Labs/EHR	162,677	61	268,886	76	164,750	66
**Outcome in 2018**	**Limitation in 2018**	**(N=302,927; 100%)**		**(N=71,322; 100%)**		**(50,774; 100%)**	
		**N**	**%**	**N**	**%**	**N**	**%**
BP control	Medical benefit, EHR	193,873	64	49,925	70	4,041	70

* In addition to continuous enrollment in medical and pharmacy benefits and KED measure exclusions, statin therapy is measured only for ages 40+ and excludes beneficiaries with cardiovascular disease, pregnancy, in vitro fertilization, clomiphene prescription, cirrhosis, and muscular pain or disease. Abbreviations: FFS, fee-for service; MA, Medicare Advantage; CKD, chronic kidney disease;

ACEi, angiotensin converting enzyme inhibitor; ARBs, angiotensin receptor blocker; SGLT2i, sodium glucose co-transporter-2 inhibitor; HbA1c, hemoglobin A1c; BP, blood pressure.

Supplemental Figure 1. KED fulfillment in 2017 compared to other measures applied to people with diabetes stratified by insurance type.

Abbreviations: MAN: medical attention for nephropathy; KED, kidney health evaluation for people with diabetes; FFS, fee-for service; MA: Medicare advantage; eGFR, estimated glomerular filtration rate (eGFR); uACR, urine albumin-creatinine ratio; HbA1c: hemoglobin A1c.

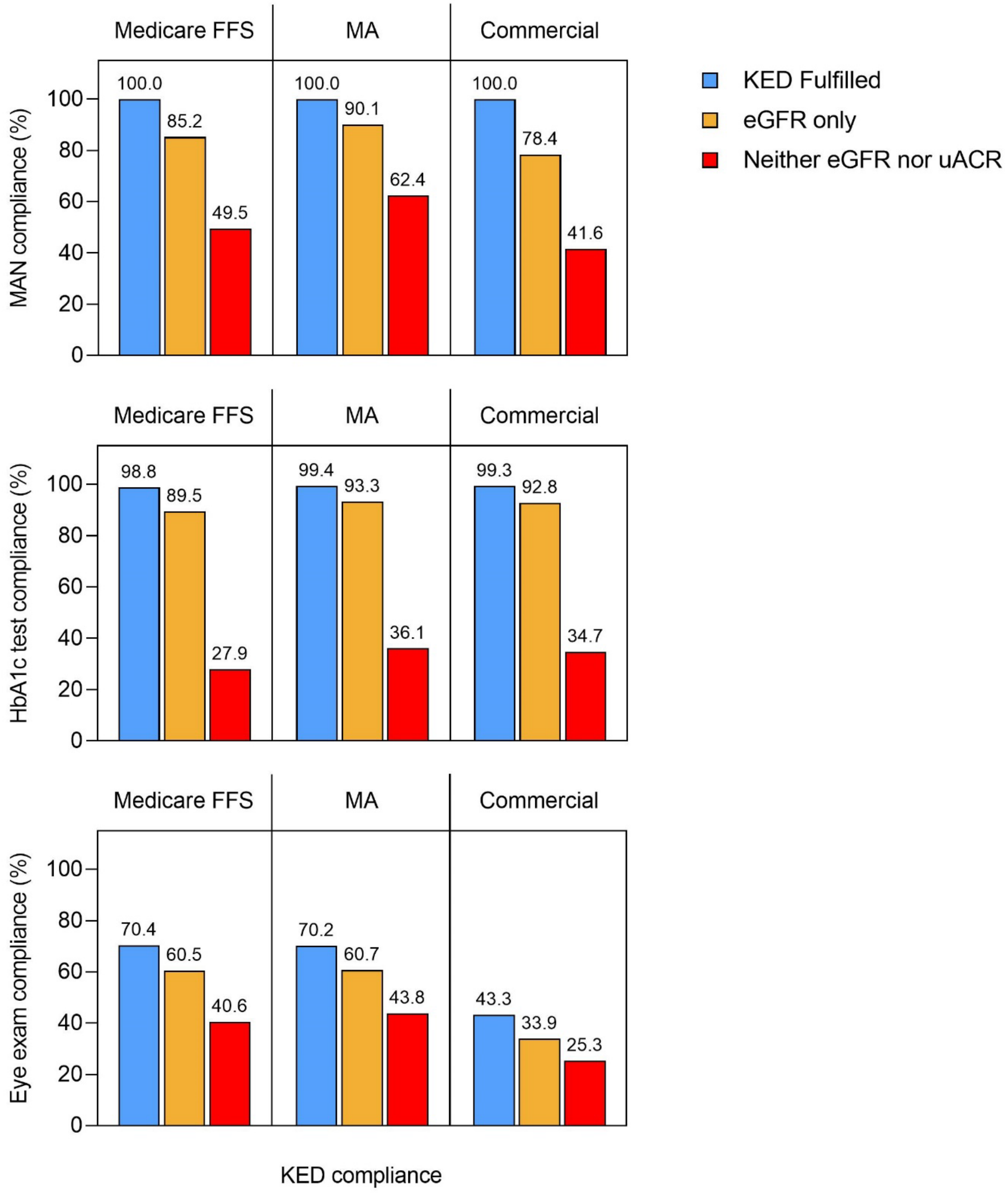

Supplement: Supplemental Data [file mmc1.pdf]
